# Autoantibodies neutralizing type I interferons in COVID-19 patients from southern Brazil

**DOI:** 10.70962/jhi.20260096

**Published:** 2026-10-06

**Authors:** Renan César Sbruzzi, Adrian Gervais, Eduarda Sgarioni, Silvia Gress, Helena Suñé Lehmen, Martina Schroeder Wissmann, Bibiana Sampaio de Oliveira Fam, Nathan A. Cadore, Mariléa F. Feira, Osvaldo Artigalás, Giovanna Câmara Giudicelli, Lucy Bizien, Aurélie Cobat, Qian Zhang, Anne Puel, Paul Bastard, Jean-Laurent Casanova, Fernanda S.L. Vianna

**Affiliations:** 1 Laboratory of Genomic Medicine, Center of Experimental Research, Hospital de Clínicas de Porto Alegre, Porto Alegre, Brazil; 2 Laboratory of Immunobiology and Immunogenetics, Universidade Federal do Rio Grande do Sul, Porto Alegre, Brazil; 3 Laboratory of Human Genetics of Infectious Diseases, Université Paris Cité, Institut Imagine, Necker Branch, INSERM UMR 1163, F-75015, Paris, France; 4 Faculty of Medicine, Federal University of Rio Grande do Sul, Porto Alegre, Brazil; 5 Medical Genetics Service, Hospital de Clínicas de Porto Alegre, Porto Alegre, Brazil; 6 Bioinformatics Core, Hospital de Clínicas de Porto Alegre, Porto Alegre, Brazil; 7 Children Research Institute, UT Southwestern, Dallas, TX, USA; 8 Pediatric Hematology-Immunology and Rheumatology Unit, Necker Hospital for Sick Children, Assistance Publique-Hôpitaux de Paris, Paris, France; 9 Laboratory of Human Genetics of Infectious Diseases, UT Southwestern Branch, Dallas, TX, USA; 10 Center for Genetics of Host Defense, UT Southwestern, Dallas, TX, USA; 11 Departments of Pediatrics and Immunology, UT Southwestern, Dallas, TX, USA

## Abstract

Inborn errors of type I IFN (IFN-I) immunity and neutralizing autoantibodies (auto-Abs) against IFN-I (AAN-I-IFNs) impair antiviral immunity and underlie critical COVID-19. Their impact on disease severity in different populations, such as Latin American populations, remains to be better understood. This study assessed the presence of AAN-I-IFNs (anti–IFN-α2, anti–IFN-ω, and anti–IFN-β) in a cohort of 458 COVID-19 patients from southern Brazil, stratified as mild (*n* = 224) versus non-mild (*n* = 234), or survivors (*n* = 369) versus deceased (*n* = 89). AAN-I-IFNs were detected in 17 patients (3.7%), with increasing prevalence across disease severity, present in one (0.4%) mild case and 16 (6.8%) hospitalized cases (P = 0.006); and eight (2.2%) survivors and nine (10.1%) deceased cases (P = 0.008). Anti–IFN-α2 and anti–IFN-ω auto-Abs were most frequently detected, while anti–IFN-β auto-Abs were found in one patient. Our findings highlight the role of AAN-I-IFNs underlying COVID-19 severity in a southern Brazilian population.

## Teaser

This study shows that neutralizing autoantibodies against type I IFNs are more frequent in severe, critical, and fatal COVID-19 cases in southern Brazil, highlighting their role in disease severity and extending global evidence to a Latin American population.

## Introduction

COVID-19 has been responsible for ∼700 million cases and 7 million deaths worldwide, including more than 700,000 deaths in Brazil alone (1). COVID-19 shows high heterogeneity in clinical presentation and severity among unvaccinated individuals, ranging from silent infection or mild presentation to critical or lethal disease. Severe COVID-19 is a systemic disorder characterized by excessive inflammatory activation and physiological dysregulation, often leading to a cytokine storm, acute respiratory distress syndrome (ARDS), and multiple organ failure (2). Advanced age is the most significant predictor of COVID-19 severity, with the risk of death doubling approximately every five years (3, *Preprint*). Additionally, men face a higher risk of death compared with women, and different comorbidities, such as diabetes, obesity, and chronic heart, lung, and kidney diseases, were also associated with worse prognosis (4, 5). The presence of comorbidities and risk factors, however, does not fully account for the interindividual variability observed in COVID-19 severity. Using an approach similar to one that has been successful for other common infectious diseases, the COVID Human Genetic Effort (CHGE) (6) aimed to discover the molecular, cellular, and immunological determinants of the severity of SARS-CoV-2 infection and highlighted the role of inborn errors of immunity (7, 8, 9), especially inborn errors of type I IFN (IFN-I)–mediated immunity, as cause of critical COVID-19 pneumonia in about 1–5% of patients (10, 11, 12).

Based on the biological mechanisms involving IFN-I–mediated immunity, the role of neutralizing autoantibodies (auto-Abs) targeting IFN-I (AAN-I-IFNs), an autoimmune phenocopy of inborn errors of IFN-mediated immunity, was hypothesized and investigated in COVID-19 patients. Large international studies led by the CHGE shown that auto-Abs neutralizing high concentrations (10 ng/ml in plasma diluted 1:10) of IFN-α2 and/or IFN-ω were present in at least 10% of unvaccinated patients with critical COVID-19 pneumonia while auto-Abs neutralizing lower concentrations, closer to physiological levels (100 pg/ml in plasma diluted 1:10), were detected in at least 13% of individuals, and auto-Abs neutralizing high concentrations of IFN-β were found in only 1% of patients (13). Such findings were later replicated in studies involving different independent cohorts worldwide (14, 15, 16, 17, 18, 19, 20, 21, 22, 23, 24, 25, 26, 27, 28, 29, 30, 31, 32, 33, 34, 35, 36, 37, 38, 39, 40, 41, 42, 43, 44, 45, 46, 47, 48, 49, 50, 51, 52, 53, 54, 55, 56, 57, 58, 59, 60), and also seen in other infectious diseases (61, *Abstract*, 62, 63). Taken together, the preexistence of these auto-Abs is the cause of ∼15% of critical COVID-19 pneumonia cases, and up to 20% of COVID-19 deaths (64, 65), being the second most relevant risk factor after old age (66). Neutralizing auto-Abs against IFN-α, IFN-β, or IFN-ω have been associated with a significantly higher risk of life-threatening COVID-19, especially when they neutralize high concentrations of both IFN-α2 and IFN-ω. Patients with these antibodies exhibited a >100-fold increased risk of severe illness compared with individuals without AAN-I-IFNs. In addition, these auto-Abs were also detected in ∼24% of a cohort of “breakthrough” hypoxemic COVID-19 pneumonia cases, despite the patients having received two doses of mRNA vaccine and developing an antibody response capable of neutralizing SARS-CoV-2 in vitro (67, 68).

AAN-I-IFNs are more frequently found in men and are rare in adults under the age of 65 years, although their prevalence increases substantially with aging. In individuals over 65 years of age, the presence of auto-Abs against IFN-ω is 0.8% (for neutralizing activity against 10 ng/ml) and 2.2% (for 100 pg/ml), reaching 1.9 and 3.82%, respectively, in those over 80 years old. For IFN-α2, the prevalence is higher, 1 and 2.6% in those over 65, and 2.4 and 4.7% after the age of 80. In younger adults, the frequency is lower, at 0.1 and 0.85% for IFN-ω at high and low concentrations, respectively. The prevalence of auto-Abs against IFN-β was lower (0.26%) and did not change at old age. The prevalence of auto-Abs neutralizing IFN-α2 increased eightfold after the age of 65, whereas auto-Abs neutralizing IFN-ω increased only 2.5-fold. In men, the increase in auto-Abs against IFN-α2 was even greater, rising more than 10-fold after the age of 65 (64). Different studies suggest that the presence of AAN-I-IFNs has genetic causes, as patients almost all with autoimmune polyglandular syndrome type 1 (APS-1) due to pathogenic variants in the *AIRE* gene produce these auto-Abs since childhood. In addition, over 75% of APS-1 patients hospitalized with severe COVID-19 pneumonia were also positive for these auto-Abs (13, 17, 66, 69). One study with two Brazilian siblings, aged 7 and 13 years, who presented with life-threatening COVID-19 pneumonia, diagnosed them with APS-1 following the detection of auto-Abs neutralizing both IFN-α2 and IFN-ω (70). To date, evidence of prevalence and impact of AAN-I-IFNs in the Brazilian population is limited to a single study that reported a 6.7% prevalence of AAN-I-IFNs in critically ill COVID-19 patients from the northeast region (71). In this study, we evaluated the presence of AAN-I-IFNs and their association with COVID-19 severity in a cohort of patients from southern Brazil.

## Results

### COVID-19 patients’ clinical and demographic description

A total of 458 COVID-19–positive patients from southern Brazil were included in the study, with clinical data and biological samples available for AAN-I-IFNs analysis and stratification by disease severity. The analyses are based on two comparisons: mild versus hospitalized (i.e., severe, critical, or fatal) and survivors versus deceased patients. Patients from both the mild and survivor groups had a median age significantly lower than those in hospitalized or deceased cases, respectively (P < 0.001, Table 1). The proportion of men increased alongside disease severity, ranging from 28.1% in the mild group to 60% in the hospitalized group (P < 0.001), and 34.4% in survivors and 59.6% in deceased cases (P < 0.001). The prevalence of diabetes and hypertension was significantly higher in the hospitalized and deceased groups compared with patients presenting with mild disease and survivors (P < 0.001). Disease severity was also associated with a higher prevalence of ARDS, intensive care unit (ICU) admission, and presence of co-infections during the disease course (P < 0.001).

**Table 1.** Clinical and demographic description of the COVID-19 cohort

​	Mild (*n* = 224)	Hospitalized (*n* = 234)	P value	Survivors (*n* = 369)	Deceased (*n* = 89)	P value
Age	42 (32–51)	60 (50–73)	<0.001	47 (37–59)	68 (58–76)	<0.001
Men	63 (28.1)	117 (50)	<0.001	127 (34.4)	53 (59.6)	<0.001
White^a^	186 (83.0)	186 (79.8)	0.402	298 (81.0)	74 (83.1)	0.381
Non-white	38 (17.0)	47 (20.2)	​	70 (19)	15 (16.9)	​
Obesity	60 (26.8)	106 (45.5)	<0.001	127 (34.5)	39 (43.8)	0.066
Diabetes	12 (5.4)	106 (45.3)	<0.001	74 (20.1)	44 (49.4)	<0.001
Hypertension	49 (21.9)	146 (62.4)	<0.001	132 (35.8)	63 (70.8)	<0.001
Chronic diseases^b^	88 (39.3)	121 (51.7)	0.009	164 (44.4)	45 (50.6)	0.178
ARDS	0 (0)	151 (64.5)	<0.001	76 (20.7)	75 (84.3)	<0.001
ICU	1 (0.4)	116 (49.6)	<0.001	56 (15.2)	61 (68.5)	<0.001
Co-infections	8 (3.6)	137 (58.5)	<0.001	75 (20.3)	70 (78.7)	<0.001

Data are presented as count (%) or median (25th–75th percentiles).

a Self-declared skin color; non-whites include individuals who self-identify as black, brown, yellow, or Native American.

b Cardiac, pulmonary, neurological, and/or rheumatic chronic diseases.

### Auto-Abs neutralizing high and low concentrations of IFN-I are more prevalent in patients with worse COVID-19 presentation and outcomes

**Figure 1.**
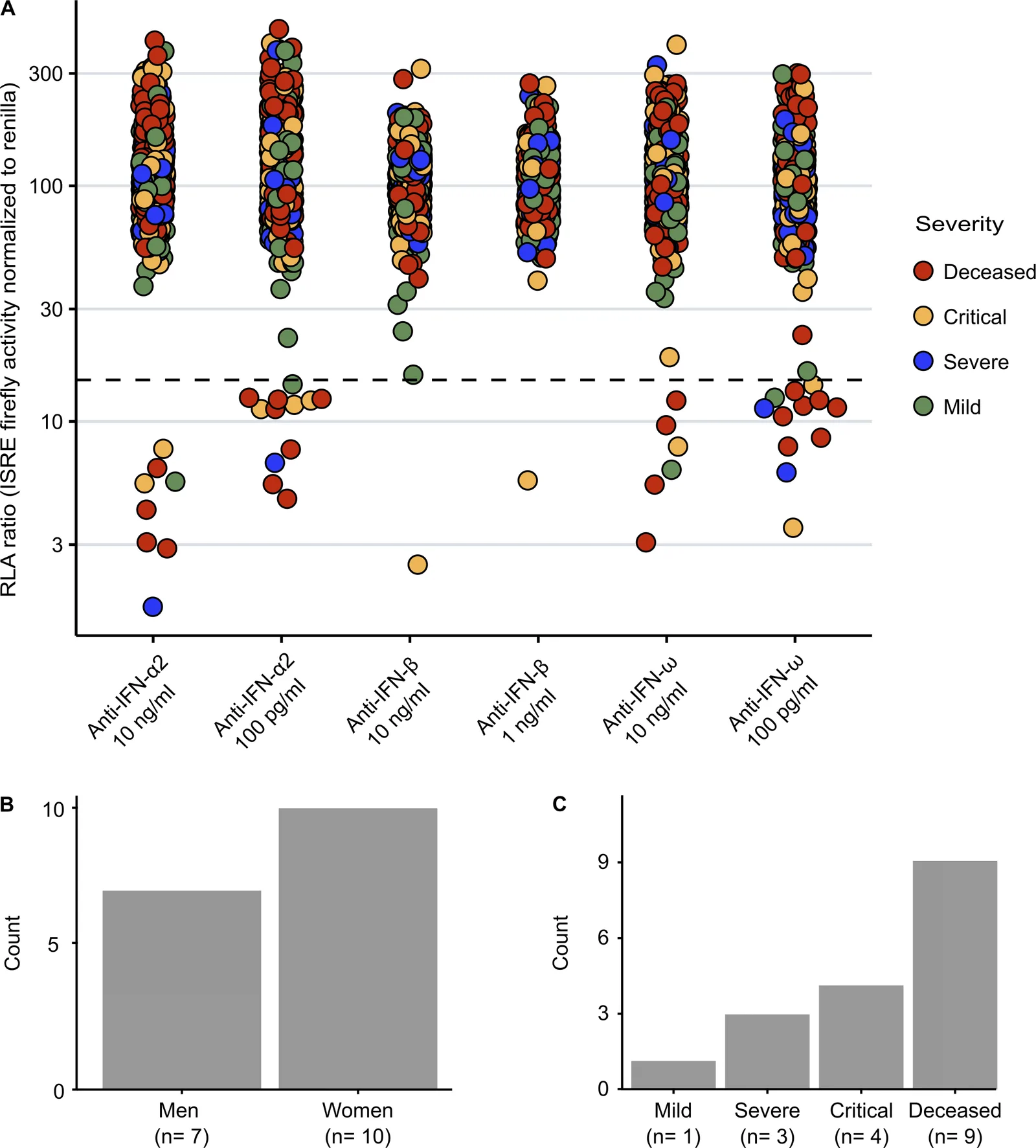
**Neutralizing auto-Abs against IFN-α2, IFN-β, and/or IFN-ω in COVID-19 patients. (A)** Results for neutralization activity of auto-Abs targeting IFN-I. Relative luciferase activity (RLA) is shown (ISRE dual-luciferase activity, with normalization against *Renilla* luciferase activity). **(B and C)** (B) Sex and (C) severity distribution amongst AAN-I-IFN–positive patients. All samples were tested once. Positive samples and samples near the threshold were tested twice. ISRE, IFN-stimulated response element.

We investigated the presence and neutralization capacity of AAN-I-IFNs using the luciferase assay and identified 17 patients (3.7% of our cohort) tested positive for auto-Abs neutralizing IFN-α2, IFN-ω, and/or IFN-β, with a higher prevalence in patients with more severe outcomes (Table 2). We identified six patients with auto-Abs neutralizing high concentrations (10 ng/ml) of both IFN-α2 and IFN-ω, four of them from the deceased group (4.5%; P4, P6, P7, and P9; Table 3), and two among survivors (0.5%; P12 and P17). No individuals in the mild group were positive for auto-Abs neutralizing high concentrations of IFN-α2 alone, whereas two hospitalized patients (0.9%; P13 and P14) tested positive for these auto-Abs. We found two patients with auto-Abs neutralizing high concentrations of IFN-α2 alone, both from the hospitalized group that survived, while auto-Abs neutralizing high concentrations of IFN-β were found in only one patient from the hospitalized group who survived (0.4%; P10). Auto-Abs neutralizing low concentrations (100 pg/ml) of IFN-α2 were found in two hospitalized patients (0.9%; P1 and P2) who died during hospitalization, and auto-Abs neutralizing low concentrations of IFN-ω were identified in four hospitalized patients, including two survivors (0.5%; P15 and P16) and two deceased (2.2%; P3 and P8) cases (Table 2 and Fig. 1 A). After adjusting for potential confounders using Firth’s bias-reduced penalized-likelihood logistic regression, AAN-I-IFNs positivity was significantly associated with increased disease severity. Patients from the hospitalized group had higher odds of testing positive for auto-Abs compared with individuals with mild disease, adjusting for age, sex, and presence of comorbidities and risk factors (age, diabetes, hypertension, chronic diseases, obesity, and male gender; odds ratio [OR] = 6.99; 95% confidence interval [CI] = 1.61–65.59; P = 0.006). The same was observed comparing deceased patients with survivors (OR = 4.36; 95% CI = 1.46–12.91; P = 0.008). Ordinal logistic regression demonstrated that the presence of AAN-I-IFNs was independently associated with increasing COVID-19 severity across the ordered clinical outcome groups (mild, severe, critical, and deceased) (adjusted OR = 5.13, 95% CI = 1.93–13.63; P = 0.001).

**Table 2.** Distribution of AAN-I-IFNs across COVID-19 severity groups

AAN-I-IFNs detected	All patients (*n* = 458)					
	Mild (*n* = 224)	Hospitalized (*n* = 234)	P value	Survivors (*n* = 369)	Deceased (*n* = 89)	P value
IFN-α2 and IFN-ω (10 ng/ml)	1 (0.4%)	5 (2.1%)	0.006	2 (0.5%)	4 (4.5%)	0.001
IFN-α2 (10 ng/ml)	0 (0%)	2 (0.9%)		2 (0.5%)	0 (0%)	
IFN-α2 (100 pg/ml)	0 (0%)	2 (0.9%)		0 (0%)	2 (2.2%)	
IFN-ω (100 pg/ml)	0 (0%)	4 (1.7%)		2 (0.5%)	2 (2.2%)	
IFN-*β* (10 ng/ml)	0 (0%)	1 (0.4%)		1 (0.3%)	0 (0%)	
IFN-α2 and IFN-ω (100 pg/ml)	0 (0%)	2 (0.9%)		1 (0.3%)	1 (1.1%)	

The same cohort of 458 patients was used for two independent comparisons: disease severity (mild vs. hospitalized) and clinical outcome (survivors vs. deceased). P values refer to the overall frequency of patients positive for AAN-I-IFNs, not to individual IFN specificities.

**Table 3.** Clinical characteristics of AAN-I-IFN–positive patients

Patient	AAN-I-IFNs detected	Age at infection	Sex	Severity	Medical history
P1	Anti–IFNα-2 100 pg/ml	48	F	Deceased	Obesity
P2	Anti–IFNα-2 100 pg/ml	52	M	Deceased	CKD; HIV; hypertension; previous tuberculosis
P3	Anti–IFN-ω 100 pg/ml	53	F	Deceased	Diabetes; hypertension; CKD
P4	Anti–IFNα-2 10 ng/ml and anti–IFN-ω 10 ng/ml	58	M	Deceased	Hypertension; obesity
P5	Anti–IFNα-2 100 pg/ml and anti–IFN-ω 100 pg/ml	63	F	Deceased	B-ALL
P6	Anti–IFNα-2 10 ng/ml and anti–IFN-ω 10 ng/ml	73	M	Deceased	Hypertension
P7	Anti–IFNα-2 10 ng/ml and anti–IFN-ω 10 ng/ml	81	F	Deceased	Hypertension; hypothyroidism
P8	Anti–IFN-ω 100 pg/ml	87	M	Deceased	COPD; hypertension; hypothyroidism
P9	Anti–IFNα-2 10 ng/ml and anti–IFN-ω 10 ng/ml	92	F	Deceased	Hypertension
P10	Anti–IFN-β 10 ng/ml	46	M	Critical	Diabetes; hypertension
P11	Anti–IFNα-2 100 pg/ml and anti–IFN-ω 100 pg/ml	47	M	Critical	None
P12	Anti–IFNα-2 10 ng/ml and anti–IFN-ω 10 ng/ml	50	F	Critical	Diabetes; hypertension; obesity
P13	Anti–IFNα-2 10 ng/ml	52	F	Critical	Diabetes; hypertension
P14	Anti–IFNα-2 10 ng/ml	64	F	Severe	Diabetes; kidney transplantation
P15	Anti–IFN-ω 100 pg/ml	65	F	Severe	Diabetes; bipolar disorder; COPD; hypertension
P16	Anti–IFN-ω 100 pg/ml	56	F	Severe	Diabetes
P17	Anti–IFNα-2 10 ng/ml and anti–IFN-ω 10 ng/ml	53	M	Mild	Secondary progressive multiple sclerosis

CKD, chronic kidney disease; B-ALL, B cell acute lymphoblastic leukemia; COPD, chronic obstructive pulmonary disease; M, male; F, female.

### Clinical characteristics of AAN-I-IFN–positive patients

Of the 17 patients with AAN-I-IFNs, 7 were men (41.2%) and 10 were women (58.8%), with a mean age of 61.2 years, with 5 (29.4%) individuals over the age of 65 years. Notably, AAN-I-IFNs were most frequently detected in deceased patients (*n* = 9, 52.9%); among survivors, the frequency was highest in critical disease (*n* = 4, 23.5%), followed by severe (*n* = 3, 17.6%), and mild cases (*n* = 1, 5.9%) (Table 2 and Table 4; and Fig. 1, A– C). Most of the patients already had comorbidities, with hypertension (47%) and diabetes (41.2%) the most prevalent. Additionally, some patients presented with specific comorbidities, such as HIV, B cell acute lymphoblastic leukemia, chronic obstructive pulmonary disease, hypothyroidism, and secondary progressive multiple sclerosis (Table 3). None of these individuals had documented in their health records any prior severe infection known to be associated with the preexistence of AAN-I-IFN with occurrence in Brazil, such as yellow fever, live-attenuated yellow fever viral vaccine disease (72), influenza A virus (73), or severe zoster infection (18, 31). Among patients positive for AAN-I-IFNs, no significant differences were observed in age, sex, ethnicity, or the prevalence of major comorbidities, including obesity, diabetes, hypertension, and chronic diseases, when comparing mild versus hospitalized disease or survivors versus deceased patients (Table 4). ARDS was the only clinical characteristic significantly associated with mortality, being present in all deceased patients compared with 50% of survivors (100 versus 50%, P = 0.029). Although hypertension and diabetes were the most frequent comorbidities among AAN-I-IFN–positive patients, their prevalence did not differ significantly between clinical outcome groups (Table 4).

**Table 4.** Clinical and demographic data across auto-Ab–positive patients

​	Neutralizing auto-Ab–positive patients (*n* = 17)					
	Mild (*n* = 1)	Hospitalized (*n* = 16)	P value	Survivors (*n* = 8)	Deceased (*n* = 9)	P value
Age	53 (53–53)	57 (50.5–71)	0.759	52.5 (47.8–62)	63 (52.5–84)	0.083
Male	1 (100%)	6 (37.5%)	0.412	3 (37.5%)	4 (44.4%)	1.000
White	1 (100%)	14 (87.5%)	1.000	8 (100%)	7 (77.8%)	0.471
Non-white^a^	0 (0%)	2 (12.5%)	​	0 (0%)	2 (22.2%)	​
Obesity	0 (0%)	4 (26.7%)	1.000	1 (14.3%)	3 (33.3%)	0.585
Diabetes	0 (0%)	7 (43.8%)	1.000	5 (62.5%)	2 (22.2)	0.153
Hypertension	0 (0%)	10 (62.5%)	0.412	4 (50%)	6 (66.7%)	0.637
Chronic diseases^b^	1 (100%)	6 (37.5%)	0.412	3 (37.5%)	4 (44.4%)	1.000
ARDS	0 (0%)	13 (81.3%)	0.235	4 (50%)	9 (100%)	0.029
ICU	0 (0%)	9 (56.3%)	0.471	5 (62.5%)	4 (44.4%)	0.637
Co-infections	0 (0%)	9 (56.3%)	0.471	3 (37.5%)	6 (66.7%)	0.347

Data are presented as count (%) or median (25th–75th percentiles).

a Self-declared skin color; non-whites include individuals that self-identify as black, brown, yellow, or Native American.

b Cardiac, pulmonary, neurological, and/or rheumatic chronic diseases.

## Discussion

In this study, we detected the presence of auto-Abs targeting IFN-I in 17 patients, comprising 3.7% of our cohort, with prevalence significantly higher amongst hospitalized cases in comparison with mild patients (OR = 6.990; 95% CI = 1.61–65.59; P = 0.006) and amongst deceased patients in comparison with survivors (OR = 4.368; 95% CI = 1.46–12.91; P = 0.008). Our analysis of AAN-I-IFNs highlights their role in disease severity and outcomes in COVID-19, as already extensively investigated and reviewed (1, 64, 74). The co-occurrence of auto-Abs neutralizing high concentrations of both IFN-α2 and IFN-ω was particularly notable in critical (1.2%) and deceased (4.5%) patients, in comparison with individuals with mild disease (0.4%). This finding aligns with previous reports that indicate that the presence of these auto-Abs is linked to an increased risk of critical illness and mortality in different populations worldwide (13, 14, 15, 16, 17, 18, 19, 21, 22, 23, 24, 25, 26, 28, 31, 33, 35, 36, 38, 40, 41, 46, 47, 48, 50, 51, 52, 53, 54, 55, 56, 57, 58, 59, 60, 64, 65, 66, 71, 75, 76, 77, 78, 79, *Preprint*, 80, 81, 82, 83, 84). However, the presence of AAN-I-IFNs is not invariably associated with severe COVID-19. For example, Meisel et al. reported that young patients with APS-1, despite harboring AAN-I-IFNs, did not develop severe COVID-19, suggesting that additional host factors, including age, may modulate disease severity (85). Consistent with this, we also identified one patient with mild COVID-19 and AAN-I-IFNs. It is important to note, in addition, that some of our deceased patients were significantly older (65, 73, 81, 87, and 92 years), and advanced age itself is associated with both increased mortality risk and a higher prevalence of AAN-I-IFNs (66). Auto-Abs neutralizing high concentrations of IFN-α2 or IFN-ω were identified mostly in critical and deceased patients, and 52.9% of patients with auto-Abs died during hospitalization, findings that also align with existing evidence that the presence of AAN-I-IFNs is a driver of severe COVID-19, particularly in older individuals (64). These findings support the investigation of neutralizing auto-Abs against IFNs-I in patients with severe or critical COVID-19 and reinforce their association with disease severity in the Brazilian population.

In this study, we analyzed 458 individuals with COVID-19 presenting with different severity and outcomes. The cohort is composed primarily of 224 mild cases (49.1%), contrasting sharply with the 63 severe (13.6%), 82 critical (17.9%), and 89 deceased (19.4%) patients. For comparative analyses, severe, critical, and deceased patients were grouped as hospitalized cases. Notably, there is a relevant median age disparity among groups, with mild patients around 42 years, while those in the hospitalized and deceased groups were significantly older, with median ages of 60 and 68 years, respectively. This age-related trend underscores the vulnerability of older individuals to severe COVID-19 and is consistent with the already known higher risk associated with older age (3). Additionally, although the cohort is predominantly composed of women (60.7%), there is an evident gender disparity as the severity of the disease increases. Specifically, the proportion of male individuals rises significantly in each progressively more severe group of patients, which is also consistent with previous findings reporting male sex as a relevant risk factor for severe COVID-19 (5). Furthermore, the prevalence of comorbidities such as obesity, diabetes, and hypertension is significantly higher in patients with more severe disease, highlighting the role of metabolic and systemic disorders in increasing the severity of the infection, as a sustained low level of immune responses reduces resistance to viral infections and previous multi-organ damage may promote the progression of COVID-19 severity (5).

Among the 17 patients positive for neutralizing AAN-I-IFNs, 10 (58.8%) were women and 7 (41.2%) were men. However, the overall prevalence of AAN-I-IFNs did not differ significantly between sexes, indicating that the higher number of women among positive individuals reflects the sex distribution of our cohort rather than a female predominance of auto-Ab positivity. We identified some important cases regarding critical COVID-19 or death in young patients with AAN-I-IFN (Table 4). P2, a 52-year-old male, and P1, a 48-year-old female, both were positive for auto-Abs neutralizing low concentrations of IFN-α2 and died during hospitalization. While P1 was obese, P2 had a complex hospital record, including previous pulmonary tuberculosis, HIV (with undetectable viral load at the time of hospitalization), and chronic kidney disease. P3, a 53-year-old female with auto-Abs neutralizing low concentrations of IFN-ω, also died during hospitalization, with diabetes, hypertension, and kidney disease as comorbidities. Similarly, P10, a 46-year-old male with auto-Abs neutralizing high concentrations of IFN-β, presented with critical disease, with only diabetes and hypertension as preexisting conditions. P4 and P6, for instance, tested positive for auto-Abs neutralizing high concentrations of both IFN-α2 and IFN-ω and died during hospitalization. Both patients were male, aged 58 and 73, and had hypertension as a relevant COVID-19 comorbidity. Together with the existing literature, our findings reinforce previous evidence linking AAN-I-IFN to an increased risk of critical illness and mortality in COVID-19 and support further investigation into the clinical utility of screening patients with critical COVID-19 to improve risk stratification and to identify individuals who may benefit from targeted therapeutic approaches, such as recombinant IFN-β (86, 87), type III IFNs (68), plasmapheresis (30), or tocilizumab (22).

Our study shows that the prevalence of AAN-I-IFN in southern Brazilian patients with critical COVID-19 (4.9% in critical, and 10.1% in deceased cases) is close to the ones observed in different cohorts, in which prevalences range from 4.2% to around 18% (13, 14, 15, 16, 17, 18, 19, 21, 22, 23, 24, 25, 26, 28, 31, 33, 35, 36, 38, 40, 41, 46, 47, 48, 50, 51, 52, 53, 54, 55, 56, 57, 58, 59, 60, 64, 65, 66, 71, 75, 76, 77, 78, 79, *Preprint*, 80, 81, 82, 84, 85). For example, Arrestier et al. reported a prevalence of 10.3% in a prospective multicenter cohort of critically ill COVID-19 patients (16), whereas Akbil et al. found a prevalence of 4.2% in a retrospective cohort (15). More recently, Staudacher et al. detected these auto-Abs in a prospective cohort of patients presenting with acute infections and showed that they were associated with worse clinical outcomes independently of SARS-CoV-2 infection (88). The variability in the reported prevalence is likely influenced by differences in study design and patient recruitment, including retrospective, prospective, and cross-sectional cohorts with distinct clinical characteristics. In addition, methodological differences between neutralization assays, particularly the IFN concentrations selected for ex vivo neutralization and the percentage of signal inhibition used to define the neutralization cutoff, may also contribute to this variability. These methodological differences should be considered when comparing prevalence estimates across studies. In Brazil, a recent retrospective study including 209 critically ill patients from the northeast region reported a prevalence of 6.7% for anti–IFN-α2 and/or anti–IFN-ω auto-Abs (71). African ancestry is more prevalent in northeastern Brazil, whereas European ancestry predominates in the southern and southeastern regions, and this disparity may explain any prevalence variability between those two populations (89). In contrast, a study from northern Brazil found prevalences of 49.0 and 21.8% in hospitalized and nonhospitalized individuals, respectively, for anti–IFN-α2 auto-Abs (90). However, this study evaluated auto-Ab reactivity rather than their neutralizing capacity, making direct comparisons with studies assessing neutralizing auto-Abs difficult and likely contributing to the differences in the reported prevalences.

One significant limitation in our study is the distribution and composition of our cohort. Unlike previous studies, our cohort included a high proportion of individuals with mild disease, which may reduce the overall chances of precisely replicating the proportion of AAN-I-IFN–positive individuals reported in other cohorts. Moreover, the age profile of our deceased, critical, and severe cases also differs considerably from earlier investigations. In our study, the mean ages for deceased, critically ill, and severe patients were 60, 57, and 69 years, respectively, compared with a mean age of ∼51 years in previously reported cohorts. Altogether, these factors likely contributed to the differences observed, underscoring the importance of cohort composition and demographic considerations in the study of AAN-I-IFNs contributions to COVID-19 severity in different populations. Another limitation is the limited sample size, with few AAN-I-IFN–positive cases, yielding wide confidence intervals. Additionally, although the patients do not have a diagnosis of any condition associated with the presence of neutralizing auto-Abs, we did not investigate any genetic etiology in the positive cases. Although we did not test for auto-Abs neutralizing the other 11 subtypes of IFN-α, it was already demonstrated that auto-Abs neutralizing IFN-α2 also usually neutralize the other 11 subtypes, which possibly leads to a higher disease risk (64, 82). Moreover, we also did not investigate the neutralizing activity of auto-Abs against glycosylated forms of IFN-α2 and IFN-ω, but a previous study reported a high rate of positive patients in with the auto-Abs neutralized both glycosylated and unglycosylated forms of IFN-α2 and IFN-ω (82). Although the association of AAN-I-IFNs with disease severity was statistically significant, the wide confidence intervals suggest some uncertainty regarding the magnitude of the effect, likely due to the small number of individuals with detectable AAN-I-IFN. Nonetheless, our findings reinforce the role of AAN-I-IFN as important determinants of COVID-19 severity and poor outcomes and highlight their potential use as prognostic biomarkers and therapeutic targets, adding evidence from a Brazilian cohort to the global context of the disease.

## Materials and methods

### COVID-19 patients

From an initial cohort designed to include 500 patients, comprising 200 individuals with mild disease and 300 with severe, critical, or deceased outcomes, this intersectional study with retrospective data review included 458 unvaccinated individuals who tested positive for SARS-CoV-2 from the Hospital de Clínicas de Porto Alegre Biobank, in the southern region of Brazil (91). Samples were collected between March 2020 and March 2021, encompassing the first wave of COVID-19 in 2020 and the beginning of the second wave in early 2021. Exclusion criteria included patients with missing clinical outcome data and inconclusive PCR detection for SARS-CoV-2. All available eligible patients were included in the study. Whole blood and plasma samples were collected and patients were stratified according to disease severity in different groups: mild group (individuals who remained asymptomatic or developed mild, self-healing, ambulatory disease with no evidence of pneumonia); severe group (patients with pneumonia requiring low-flow oxygen [<6 liters/min]); critical group (patients with critical disease requiring mechanical ventilation [continuous positive airway pressure, bilevel positive airway pressure, intubation, or high-flow oxygen over 6 liters/min oxygen], septic shock, or multi-organ damage requiring admission in the ICU); and a group of individuals who died due to COVID-19 pneumonia. The 458 patients were stratified according to two different clinical classifications: disease severity (mild [*n* = 224] versus hospitalized [*n* = 234], including severe, critical, and fatal cases) and clinical outcome (survivors [*n* = 369] versus deceased patients [*n* = 89]). The research was approved by the Research Ethics Committee of Hospital de Clínicas de Porto Alegre (CAAE: 36974620.3.0000.5327).

### Luciferase assay for neutralizing auto-Abs detection

The presence and blocking activity of anti–IFN-α2, anti–IFN-ω, and anti–IFN-β neutralizing auto-Abs were measured using a luciferase assay. Briefly, HEK293 T cells were transfected with a plasmid containing the firefly luciferase gene under the control of the human IFN-stimulated response element promoter in the pGL4.45 backbone and a plasmid constitutively expressing *Renilla* luciferase (pRL-SV40), used for luminescence data normalization. Cell transfection was performed using X-tremeGene9 transfection reagent (ref. number 6365779001; Sigma-Aldrich) for 24 h. Cells in DMEM (Thermo Fisher Scientific) supplemented with 2% FCS and 10% healthy control or patient serum/plasma (after complement inactivation at 56°C, for 20 min) were either left unstimulated or were stimulated with IFN-α2 (ref. number 130-108-984; Miltenyi Biotec) or IFN-ω (ref. number SRP3061; Merck) at 10 ng/ml or 100 pg/ml, or with IFN-β (ref. number: 130-107-888; Miltenyi Biotec) at 10 ng/ml or 1 ng/ml for 16 h at 37°C. Cells were then lysed, and luciferase levels were measured with the Dual-Luciferase Reporter assay system (ref. number E1980; Promega) according to the manufacturer’s protocol. Luminescence intensity was measured with a VICTOR-X Multilabel Plate Reader (PerkinElmer Life Sciences), and firefly luciferase activity values were normalized against *Renilla* luciferase activity values. These values were then normalized against the median level of induction for non-neutralizing samples and expressed as a percentage. Samples were considered neutralizing if luciferase induction after normalization against *Renilla* luciferase activity was below 15% of the median value for controls tested the same day (65). Analysts were blinded to clinical outcomes during experimental assays.

### Statistical analysis

Continuous data are shown as mean ± standard deviation or median (25th–75th percentiles). Categorical variables are reported as number (percentage), percentage, or relative frequency. The Kolmogorov–Smirnov test was used to verify the normality of continuous variables. Continuous data were compared between groups by Kruskal–Wallis test, followed by the Dunn post hoc test. Categorical variables were compared between the groups by Pearson’s χ^2^ test and Fisher’s exact test, and odds ratios and 95% confidence intervals were estimated by Firth’s bias-reduced penalized-likelihood logistic regression, using the logistf package. To evaluate the association between neutralizing auto-Abs and the ordered spectrum of COVID-19 severity (mild, severe, critical, and deceased), an adjusted ordinal logistic regression model (proportional odds model) was fitted using the rms package. All analyses were performed using the Statistical Package for the Social Sciences software, version 20.0 (SPSS Inc.) and R software (R Core Team).

### Online supplemental material

Table S1 shows normalized RLA values for auto-Abs–positive patients.

## Supplementary Material

Table S1 shows normalized relative luciferase activity of anti–IFN-I auto-Ab–positive patients.

## Data Availability

The original contributions presented in the study are included in the article/supplementary materials, and further inquiries can be directed to the corresponding author.
