# Supplementary material for: Autoantibodies neutralizing type I interferons in COVID-19 patients from southern Brazil
Source: J Hum Immun. 2026 Oct 6;2(6):e20260096. doi: 10.70962/jhi.20260096 (PMC13641011; doi:10.70962/jhi.20260096)
Supplement: Supplementary file 1 [file jhi_20260096_tables1.docx]

**SUPPLEMENTARY MATERIAL 1**

**From “AUTOANTIBODIES NEUTRALIZING TYPE I INTERFERONS IN COVID-19 PATIENTS FROM SOUTHERN BRAZIL”**

Renan C. Sbruzzi *et al*.

**Corresponding author:** Fernanda S. L. Vianna

**E-mail address:** [fvianna@hcpa.edu.br](mailto:fvianna@hcpa.edu.br)

**The file includes:** Supplementary Table 1 (S1)

**Supplementary Table 1**. Normalized relative luciferase activity of anti-IFN-I auto-Abs positive patients.

|  | **Normalized relative luciferase activity** | | | | | |
| --- | --- | --- | --- | --- | --- | --- |
| **Patient** | **Anti-IFN-α2 10 ng/mL** | **Anti-IFN-α2 100 pg/mL** | **Anti-IFN-ω**  **10 ng/mL** | **Anti-IFN-ω 100 pg/mL** | **Anti-IFN-β**  **10 ng/mL** | **Anti-IFN-β**  **1 ng/mL** |
| P01 | 169.373455 | **11.2893533** | 239.30518 | 23.2737507 | 130.232428 | 142.922496 |
| P02 | 100.305895 | **7.60781369** | 93.8847088 | 65.912243 | 99.1019981 | 90.1638879 |
| P03 | 122.296264 | 112.025085 | 54.5609516 | **7.82734193** | 121.459486 | 95.6726218 |
| P04 | **3.067588** | **12.3809099** | **9.63452289** | **13.4561083** | 122.677532 | 146.048905 |
| P05 | 154.400548 | **5.41734342** | 257.065354 | **12.3185111** | 170.951416 | 184.082988 |
| P06 | **2.89331603** | **12.4732057** | **12.2670589** | **8.52660102** | 88.7893545 | 172.25466 |
| P07 | **6.34532796** | **4.69589544** | **5.39539847** | **11.4653768** | 177.894334 | 152.304706 |
| P08 | 180.160879 | 210.961496 | 171.238043 | **11.6584633** | 119.232458 | 118.33032 |
| P09 | **4.38429568** | **12.6047525** | **2.89331603** | **10.5151435** | 173.011439 | 165.073505 |
| P10 | 205.602751 | 190.45482 | 267.168005 | 133.468507 | **2.4678729** | **5.62042435** |
| P11 | 305.360013 | **12.2670589** | 302.620942 | **3.53831266** | 148.76195 | 159.829737 |
| P12 | **7.65144108** | **11.2849193** | **7.82219831** | **14.2734621** | 67.6892927 | 152.094298 |
| P13 | **5.46128743** | **11.7639983** | 110.698133 | 73.481269 | 110.698133 | 129.464434 |
| P14 | **1.63422173** | **6.67677761** | 72.9042356 | 54.5609516 | 98.2540672 | 89.2290026 |
| P15 | 75.0861059 | 106.150807 | 85.2062855 | **11.8057222** | 128.79653 | 97.2873194 |
| P16 | 100.093236 | 113.443551 | 111.304932 | **6.07676798** | 141.431695 | 141.644045 |
| P17 | **5.76643631** | **14.3701737** | **6.23716329** | **12.6106154** | 86.7914471 | 86.2346133 |

Bold values indicate auto-abs positive RLAs.
